# Spatio-temporal characteristics of population responses evoked by microstimulation in the barrel cortex

**DOI:** 10.1038/s41598-018-32148-0

**Published:** 2018-09-17

**Authors:** Shany Nivinsky Margalit, Hamutal Slovin

**Affiliations:** The Gonda Multidisciplinary Brain Research Center, Bar-Ilan University, Ramat Gan, 52900 Israel

## Abstract

Intra-cortical microstimulation (ICMS) is a widely used technique to artificially stimulate cortical tissue. This method revealed functional maps and provided causal links between neuronal activity and cognitive, sensory or motor functions. The effects of ICMS on neural activity depend on stimulation parameters. Past studies investigated the effects of stimulation frequency mainly at the behavioral or motor level. Therefore the direct effect of frequency stimulation on the evoked spatio-temporal patterns of cortical activity is largely unknown. To study this question we used voltage-sensitive dye imaging to measure the population response in the barrel cortex of anesthetized rats evoked by high frequency stimulation (HFS), a lower frequency stimulation (LFS) of the same duration or a single pulse stimulation. We found that single pulse and short trains of ICMS induced cortical activity extending over few mm. HFS evoked a lower population response during the sustained response and showed a smaller activation across time and space compared with LFS. Finally the evoked population response started near the electrode site and spread horizontally at a propagation velocity in accordance with horizontal connections. In summary, HFS was less effective in cortical activation compared to LFS although HFS had 5 fold more energy than LFS.

## Introduction

Electrical stimulation has long been an important tool for exploring the organization and function of the nervous system as well as an important communication channel for brain machine interfaces (BMI). For over a century, scientists have used different applications and protocols of electrical stimulation to artificially activate brain regions and investigate their functionality and connectivity^1^. Moreover, electrical stimulation enabled breakthrough advances in many clinical applications based on BMI, for example artificial cochlea can restore hearing to deaf patients and in the basal-ganglia it can alleviate motor impairment of parkinsonian patients, reduce chronic neuropathic pain and recently it was found to be a useful treatment in depression^2–5^.

Intra-cortical microstimulation (ICMS) is a widely used electrical stimulation technique where short pulses of relatively low amplitude currents (in the range of µA) are delivered to the cortical tissue via a small microelectrode tip and induce the excitation of nearby cell bodies and axons^6,7^. This method has played a central role in experimental neuroscience and helped providing a causal link between neuronal activity and cognitive or motor functions^8,9^ and it was used for functional mapping of various brain areas, e.g. the motor cortex^10–12^, frontal eye field area^13,14^ etc. In addition, it was investigated in the visual^15,16^, auditory^17–19^ and somatosensory regions^20,21^, revealed functional connectivity between different regions in the brain^22–25^ (for reviews see^1,9,26^) and was shown to affect sensory perception, behavioral responses and behavioral decisions^27^.

The effects of ICMS on neural activity depend on the stimulation parameters, which include pulse duration, current amplitude, train duration and stimulation frequency^26^. While some studies investigated the effects of frequency, current and/or amplitude on behavioral performance, psychophysical curves^1,28–30^ or the generation of saccadic eye movement^31^, fewer studies investigated and measured the *direct* effect of these parameters on the evoked spatio-temporal patterns of the neural activity^32–34^. The effect of stimulation frequency on the evoked spatio-temporal patterns of neuronal activity is largely unknown. Studies using deep brain stimulation (DBS)^35^ suggested that low frequency electrical stimulation (LFS; ranging between 0.5–25 Hz for this technique) induce depolarization of neuronal membranes and evokes action potentials, whereas higher frequencies stimulation (HFS), ranging up to 30 kHz (depends on the brain region that is stimulated and the application technique) resulted in inhibition of action potentials in both central^35,36^ and peripheral neurons^37,38^.

Therefore, the effect of low and high frequency ICMS on the spatio-temporal patterns of the evoked neural response are not well understood. To investigate this issue we inserted a microelectrode to the barrel cortex, a well-studied brain area, in anesthetized rats. We then stimulated the cortical tissue using 3 different ICMS conditions: single-pulse stimulation, high-frequency stimulation (HFS, 500 Hz) and a lower frequency stimulation (LFS, 100 Hz). The latter frequency is within the range of stimulation frequencies that were widely used in many previous ICMS studies and was shown to be highly effective in driving the neural activity and informative in characterizing the evoked neural and behavioral responses^39–43^. Using voltage-sensitive dye imaging (VSDI) we then imaged the population responses in the stimulated area at high spatial (mesoscale) and temporal resolution (Shoham *et al*., 1999; Slovin *et al*., 2002).

Our results show that LFS and HFS evoked population responses with distinct spatio-temporal characteristics. We found that ICMS at LFS was more effective in cortical activation compared with ICMS at HFS: cortical activation extended over a larger region and evoked higher neural response. In addition, we found that the two stimulation conditions induced neural activity near the stimulating electrode that propagated laterally over the cortical surface. The propagation velocity of the evoked pattern suggests the involvement of horizontal connections in lateral propagation.

## Results

**Figure 1:**
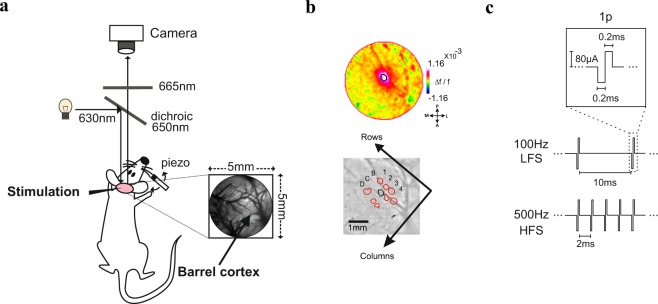
ICMS combined with voltage-sensitive dye imaging in the barrel cortex. (**a**) A schematic diagram of the VSDI setup combined with ICMS in the barrel cortex. (**b**) Top: VSD response map at 10 ms post C2 whisker deflection (see Methods). The black contour denotes the barrel field position and outlines 75% of peak VSD response. Bottom: The contours of different barrel fields obtained using different whiskers’ stimulation. The contour of C2 barrel field shown on the top is marked in black. The black arrows denote the axis for rows and columns in the barrel cortex. (**c**) Schematic illustration of the used ICMS parameters in the different conditions: 1p, LFS, HFS.

We measured neuronal population responses evoked by intra-cortical microstimulation (ICMS) in the upper layers (L2/3) of the barrel cortex of anesthetized rats, using voltage-sensitive dye imaging (VSDI) which enabled us to image neural activity at high spatial (mesoscale, 50^2^ µm^2^/pixel) and high temporal resolution (100 Hz; see Methods) simultaneously (Fig. 1). The fluorescence dye signal from each pixel reflects the sum of membrane potential from all neuronal elements (dendrites, axons and somata) and therefore is a population signal (rather than response of single neurons). In addition, the VSD signal emphasizes subthreshold synaptic potentials (but reflects also suprathreshold activity^44–46^).

**Figure 2:**
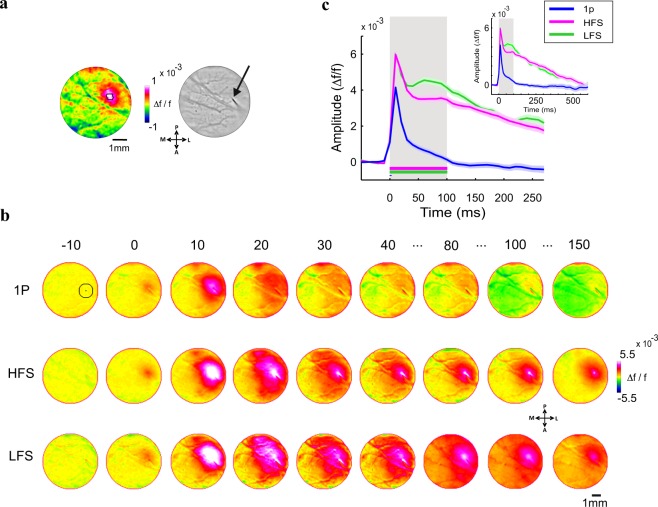
Spatio-temporal activation patterns evoked by ICMS in the barrel cortex. (**a**) Left: VSD response map (∆f/f, fluorescence levels) evoked by C2 whisker deflection, 10 ms post stimulation onset (1 pulse, 50 ms duration). The black contour outlines 75% of peak response. Fluorescence level is depicted by the color bar. Right: the microelectrode position (black arrow), over the blood vessels pattern of the barrel cortex. (**b**) Population response maps evoked by ICMS in the barrel cortex, an example session. The numbers above the maps show the time in ms after ICMS onset. The circle ROI (radius = 0.55 mm, black contour) centered on peak response is depicted on the upper row, left map. Maps are color coded. (**c**) Time course of the VSD signal from the example sessions in b. The VSD signal was averaged over the ROI pixels depicted in b. Shaded area is ± 1 SEM over trials (1p, n = 15; HFS, n = 15; LFS, n = 11 trials). Inset: The VSD response at longer time scale, showing the response decay back to baseline. Shaded rectangle represents the ICMS duration for LFS and HFS conditions.

At the beginning of each experiment, a single whisker was deflected on one side of the animal’s whisker pad, while we imaged the evoked response in the contralateral barrel cortex. The early evoked population response pattern revealed the location of the barrel field of the stimulated whisker, in the imaged area (Fig. 2a left; VSD signal is measured as fluorescence change (∆f/f), map is color coded). We used the evoked activation pattern in order to direct the microelectrode to the barrel cortex (Fig. 2a right) and insert it into the upper layers (250–400 µm). We then stimulated the barrel cortex with biphasic square pulses, current amplitude of 80 µA (see Methods) with the following parameters (Fig. 1c): single-pulse stimulation (1p), low-frequency stimulation (LFS; 100 Hz; 10 pulses, 100 ms duration) and two high frequency conditions of different duration lengths: (i) 100 ms of high-frequency stimulation (HFS; 500 Hz; 50 pulses), in this condition the stimulation length equals the LSF, but it has 5 fold more energy. (ii) 20 ms of high-frequency stimulation (HFS short; 500 Hz; 10 pulses), in this condition, the amount of energy equals to that of LFS, but stimulation length is much shorter. As the VSD signal was sampled at 100 Hz, any temporal differences, smaller than 10 ms, between the different ICMS conditions will not be detected by the current system.

### Population responses evoked by ICMS

Figure 2b shows data from an example session: a sequence of VSD maps evoked by ICMS for 1p (top row), HFS (middle row) and LFS (bottom row). The VSD response, appeared around the electrode tip, starting to increase already within the ICMS onset frame (t = 0 ms). In the subsequent time frames (each frame is 10 ms duration) the population activity quickly spread over the barrel cortex in an anisotropic manner, as previously reported^47,48^. The anisotropic VSD response showed a larger spread along the rows of the barrel cortex than across the columns as previously reported^48–50^ (see Supplementary Fig. S1). The maps show that HFS and LFS evoked higher neuronal population response than 1p stimulation, as expected (see maps at 10–30 ms), while at later times the response evoked by LFS activated a larger region (e.g. more red/pink pixels in the maps at 80–100 ms) compared with the HFS condition (although HFS contains 5 fold more energy than LFS). To compute the time course of the VSD response we defined a circular region of interest (ROI) that was centered on the peak cortical response (black circular contour in Fig. 2b, upper row, left map; see Methods). The VSD time course averaged across the ROI pixels is shown at Fig. 2c (same example session as in Fig. 2b). The responses in all conditions showed a fast and narrow peak activation and as described above, the peak responses for LFS and HFS were higher than the response to 1p (4.3 × 10^−3^ ± 1.12 × 10^−4^ (mean ± sem); 6.1 × 10^−3^ ± 2.24 × 10^−4^ and 6.2 × 10^−3^ ± 1.25 × 10^−4^ ∆f/f for 1p (n = 15 trials) HFS (n = 15) and LFS (n = 11) respectively; p < 0.001, Wilcoxon rank sum test, Bonferoni corrected). Interestingly, for the longer ICMS conditions, the VSD signal showed a more sustained response (40–100 ms) that was higher for the LFP condition (Fig. 2c green curve) compared with the HFS condition (Fig. 2c pink curve). Finally, after the stimulation was ended, all VSD responses showed a slow descending phase to baseline which was much faster for 1p than for HFS and LFS conditions (Fig. 2c inset).

**Figure 3:**
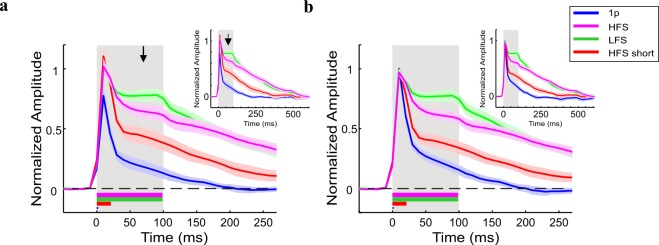
Normalized time course of VSD response evoked by different ICMS conditions. (**a**) Grand average normalized time course. The VSD time course was computed over a ROI in each recording session and then normalized to peak response of the LFS condition. Next, the time course of the VSD response for each condition was averaged across all sessions. The arrow indicates the first time point where the LFS curve deviated significantly from the HFS (t = 70 ms; Wilcoxon rank-sum test: p < 0.05). The shaded area is ± 1 SEM over sessions. Horizontal colored bars refer to ICMS duration. (**b**) Peak normalized time course of the VSD signal, averaged across sessions (on each condition the VSD response was normalized to its own peak and then averaged across sessions). Shaded area represents ± 1 SEM over sessions (1p, n = 6; HFS, n = 6; LFS, n = 6; HFS short, n = 6 sessions). Inset: The VSD response at longer time scale, showing the response decay back to baseline. Shaded rectangle represents the ICMS duration for LFS and HFS.

Next we computed the grand analysis of the VSD signal time course for the different stimulation conditions (Fig. 3a). To account for variance of the VSD response across recording sessions (resulting from variance across animals, VSD staining quality etc.), on each imaging session the VSD signals in the different conditions were normalized to peak response of the LFS condition. Next, the VSD responses were averaged across all sessions. The red curve depicts the normalized grand analysis time course for the HFS short condition (10 pulses; 500 Hz) that has equal energy as LFS and therefore has shorter time duration (20 ms). The grand analysis confirmed the main observations shown in the example session: narrow and fast peak activation for all conditions. Although the peak VSD response was higher for HFS and LFS than the peak response for the 1 P, it was not statistically significant across sessions (p > 0.1, Wilcoxon rank sum test, Bonferoni corrected; 0.77 ± 0.09 (mean ± sem); 1.05 ± 0.04; 1 ± 0 and 1.12 ± 0.05 for 1p (n = 6) HFS (n = 6 sessions) LFS (n = 6) and HFS short (n = 6) respectively). Next, a more sustained VSD response appeared for the longer stimulation conditions, and it was higher in the LFS compared with the HFS condition (p < 0.05 at 70–100 ms; Wilcoxon rank sum test; black arrows in Fig. 3a), although HFS has 5 fold more energy than LFS (50 vs. 10 pulses, respectively). This result is consistent with a previous study who showed that the HFS suppressive effect is independent upon stimulus duration^36^. At the end of stimulation, the VSD responses in all conditions showed a slower descending phase that was shorter for the 1p compared with all other conditions (Fig. 3a inset).

The time to peak response was similar for all stimulation conditions (11.6 ± 1.66 ms for HFS, LFS and HFS short and 10 ± 0 ms for 1p stimulation). The normalized peak response amplitude was similar for HFS and HFS short as expected because in both conditions the peak response appeared following ~8 pulses of stimulation (Fig. 3a; 1.12 ± 0.05 and 1.05 ± 0.04 for HFS short (n = 6) and HFS (n = 6) p > 0.05; Wilcoxon rank sum test). Interestingly, the normalized peak response of LFS was also similar and not statistically different form the peak response of HFS (p = 0.36; Wilcoxon rank sum test) despite the fact that it occurred after only ~2 pulses of stimulation.

To further investigate the temporal characteristics of HFS and LFS evoked responses we normalized the VSD time courses to the peak response of each ICMS condition (Fig. 3b). Similar to shown in Fig. 3a, a sustained VSD response appeared for the longer stimulation conditions, and it was higher in the LFS compared with the HFS condition (p < 0.05 starting at 40 ms; Wilcoxon rank sum test). Although the time to peak response was similar among all ICMS conditions (see above), the response dynamics following the end of stimulation showed variation among the conditions (Fig. 3b, and inset). The time for the response to decline *below* half peak response was longer for HFS short relative to 1p (30 ± 2.6 and 48.33 ± 16.4 ms, for 1p (n = 6) and HFS short (n = 6) respectively), however it was not significantly different (p = 0.48; Wilcoxon rank sum test). Similarly, the LFS response showed a longer time to decline below half peak response compared to the HFS condition but this was not significant (175 ± 17.1 and 143.33 ± 24.7 ms, for LFS (n = 6) and HFS (n = 6) respectively; p = 0.1; Wilcoxon rank sum test). However, the time for the response to decline below half peak response was significantly longer for the LFS and HFS than 1p stimulation (p < 0.01; Wilcoxon rank sum test, Bonferoni corrected) and HFS short (p < 0.05 for LFS. p = 0.09 for HFS; Wilcoxon rank sum test, Bonferoni corrected). In summary, the LFS condition evoked a higher neural activation during the sustained response, with comparison of both HFS conditions (short and long train). Thus the HFS condition was inferior to the LFS in activating neuronal populations.

### Characteristics of the spatial pattern

The ICMS conditions can be divided into two stimulation groups: iso-energy but with different duration (LFS, HFS short) and iso-duration but with different energy (LFS, HFS). As for the iso-energy group, because the LFS duration was longer than HFS (100 and 20 ms, respectively) it is possible that stimulation duration (rather than frequency) affected the lower evoked activity of the HFS condition. Therefore, below we focus on ICMS conditions with similar durations.

**Figure 4:**
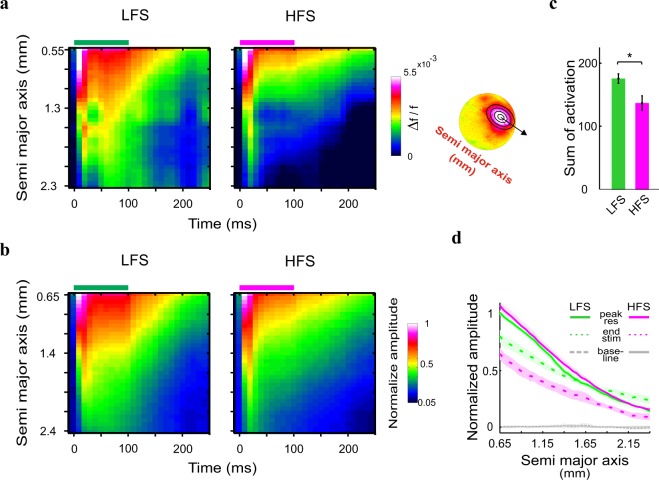
Space time analysis using elliptical ring ROIs. (**a**,**b**) Space vs. time plots: the VSD response at increasing distances from the center as function of time. Response in each ellipse was normalized to the mean peak response of the five first ellipses in the LFS condition (see Methods). Horizontal bars at the top represent the stimulation duration for an example session (a left; same as at Fig. 2) and for grand average (**b**). a right: Schematic illustration of elliptical ring shape ROIs. Successive ellipses are increased by one pixel (50 μm). Y-axis represents the size of the ellipses semi major axis. (**c**) Sum of response activation over space and time (from t = 0 to t = 100 ms), for the maps depicted in b. Error bars represents ± 1 SEM over sessions. Wilcoxon rank-sum test: *p < 0.05. (**d**) Spatial profile of the responses, across all sessions, at time of peak response (peak res, solid line) and when stimulation was ended (end stim, dashed line). Gray lines represent the baseline activity for HFS (solid line) and LFS (dashed line). The profile is normalized to the mean peak response across the five first rings of LFS. The shaded area is ± 1 SEM over sessions (LFS, n = 6; HFS, n = 6 sessions).

As shown in Fig. 2b, the population response evoked by ICMS appeared first around the microelectrode tip and then propagated horizontally, over cortical surface in an anisotropic manner (see Supplementary S1). To quantify and compare the profile of the spatial spread for HFS and LFS, we applied an elliptical ring shape ROI analysis (see Methods). We generated a continuous set of non-overlapping elliptical rings (see schematic illustration in Fig. 4a right), that were fitted to the evoked response pattern at 10 ms post stimulation. Figure 4a left shows the space-time maps for an example session and Fig. 4b shows the grand analysis of the space-time maps, where the VSD signal was normalized to the peak response of the LFS condition. The maps show the neuronal response as function of distance along the semi major axis of the fitted ellipses from the central ellipse (y-axis; see Methods) for each time point (x-axis). Thus, using this approach we could investigate the VSD signal propagation from the center of response to adjacent cortical regions. The spatio-temporal profile of the HFS condition in the example session (Fig. 4a) and in the grand analysis (Fig. 4b) showed less activation over time and space compared with the LFS condition. To quantify this difference, we computed the sum of overall activation during stimulation (i.e. 0–100 ms from stimulation onset) and over space (up to mean semi major axis: 2.4 ± 0.1 mm) for each session. Figure 4c depicts the summed normalized activation, which is significantly higher for the LFS than the HFS (173.74 ± 8.9 and 136.17 ± 11.75 for LFS (n = 6) and HFS (n = 6) respectively; p < 0.05, Wilcoxon rank sum test). This result supports the assumption that high frequency stimulation is less effective in activating neuronal responses relative to lower frequency stimulation. Figure 4d shows the spatial profile for LFS and HFS at peak response (continuous lines) and at time of stimulation end (t = 100 ms; dashed lines). At peak response, the spatial profile of the evoked activity for the two conditions is similar, however when stimulation is ended, the spatial profile of the LFS condition is significantly higher than HFS (p < 0.001, Wilcoxon rank sum test). Both spatial profiles show a significant deviation from baseline activity (i.e. activity before ICMS onset; gray lines) at time of peak response as well as the end of stimulation (Fig. 4d). When investigating the deviation of LFS spatial profile from HFS spatial profile at later time i.e. at 200 ms post stimulation, the LFS was higher from HFS (and baseline) at remote distances (1.95–2.4 mm on the semi major axis; p < 0.05, Wilcoxon rank sum test). Together, these results suggest that the LFS was superior in activating cortical populations relative to HFS. LFS generated higher neuronal responses, extending over larger cortical space during ICMS stimulation and in addition, the VSD response showed a slower decay to baseline relative to HFS, at regions remotely located from the center of response. Similar results were obtained for a circular ring shape ROI centered on peak activation in space (see Supplementary Fig. S2).

Although LFS was more effective in cortical activation relative to HFS, it is possible that a lower frequency condition, i.e. 50 Hz will be even more effective. To test this option, we stimulated the barrel cortex with a lower frequency of 50 Hz. Figure S3 shows the normalized VSD response to 100 ms stimulation at 50 Hz (5 pulses; 80 µA) and 100 Hz (LFS) and spatial spread, normalized to peak response of LFS condition. There are no significant differences between the LFS and the 50 Hz stimulation.

### The propagation of the VSD response over space

**Figure 5:**
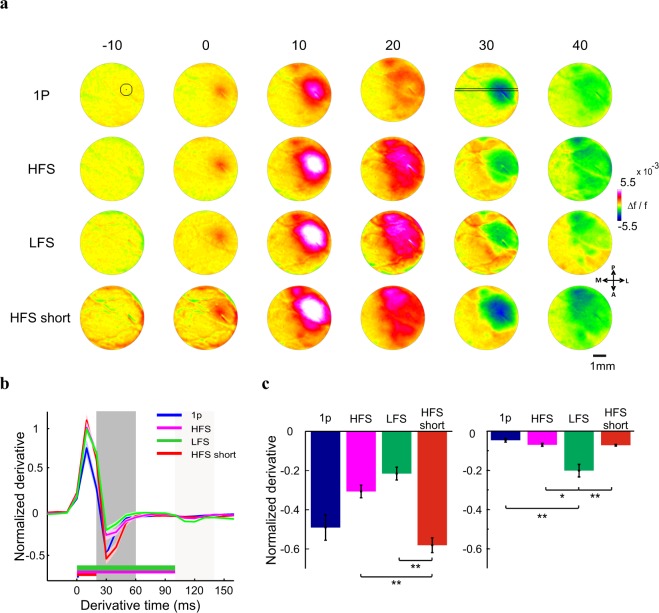
Derivative maps and analysis. (**a**) A sequence of derivative VSD maps following ICMS. Derivative was computed by subtracting the VSD maps obtained at t-20 ms from maps obtained at t. The numbers above the maps are thus corresponding to the derivative time (t = t-20). Top row: circle ROI (map at t = −10) appears on peak VSD response in space. The black lines (map at t = 30) denotes a horizontal spatial cut crossing the peak VSD response in space (see spatial profile analysis appears in Fig. 6). (**b**) Normalized derivative response averaged across sessions (the VSD response was normalized to LFS condition peak response). The dark shaded area represents the time window for the analysis of min derivative at early times (20–60 ms after ICMS onset) while the lighter shaded area represents the time window analysis for later times (100–140 ms). (**c**) Trough of negative derivative for each conditions at early times (left, 20–60 ms) and late times (right, 100–140). Values were averaged across sessions, error bars are one SEM over sessions (1p, n = 6; HFS, n = 6; LFS, n = 6; HFS short, n = 6 sessions). Wilcoxon rank-sum test: *p < 0.05 **p < 0.01.

ICMS evoked neuronal population response that showed a fast spread of activity over few mm in the cortex, we therefore decided to study the spatio-temporal dynamics of this signal propagation. We computed the derivative maps of the VSD responses: from each VSD map we subtracted the VSD map measured 20 ms earlier. Figure 5a depicts a sequence of derivative maps from an example session. The maps show an early (0–10 ms) positive change of activation corresponding to increased population response around the electrode site upon ICMS onset, in all conditions. Later times (30–40 ms; Fig. 5a) show negative derivative values, corresponding for the fast descending phase of the VSD signal after arriving to peak response (see Fig. 3a,b for the time course of the VSD signal). This is further shown in the grand analysis of the derivative time course (Fig. 5b; The VSD signal was normalized to peak response of the LFS condition). Derivatives were computed for a circular ROI (Fig. 5a, top left map) and then averaged across all sessions. The negative derivatives values are significantly larger for HFS short than LFS or HFS (p < 0.001, Wilcoxon rank sum test, Bonferoni corrected; Fig. 5c left). The min negative derivative of all stimulation conditions are significantly smaller than zero (p < 0.05, sign-ranked test for a significant difference from zero) and all appeared at similar time regardless of stimulation length (30 ± 0 ms for 1p, 31.6 ± 1.6 ms for HFS short and HFS and 33.3 ± 2.1 ms for LFS; p > 0.5 Wilcoxon rank sum test). The LFS condition showed another trough of negative derivative at much later times, corresponding to the end of stimulation (100–140 ms; Fig. 5b,c right). This negative phase is significantly larger for LFS than all other conditions (*p < 0.05, **p < 0.01, Wilcoxon rank sum test, Bonferoni corrected). Together these results show that the major positive and negative derivative components appear in all conditions.

**Figure 6:**
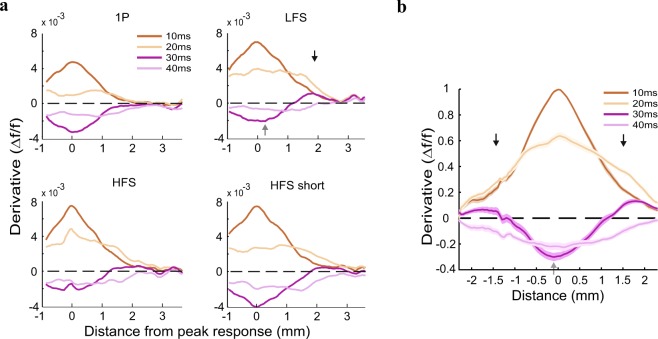
Spatial profiles of the derivative maps. (**a**) Spatial profiles as function of time, from the example session shown in Fig. 5 (spatial profile cut is depicted in Fig. 5a, upper row, map at t = 30). (**b**) Grand average normalized spatial profile, across HFS, LFS and HFS short conditions (HFS, n = 6; LFS, n = 6; HFS short, n = 6 sessions). On each session the VSD signal was normalized to peak response of each profile at t = 10 ms. The shaded area is ± 1 SEM over sessions.

Our next step was to study the spatial profile of the derivative maps. For this purpose we used a horizontal spatial profile (Fig. 5a, top row, map t = 30 ms; see Methods). Figure 6a shows the spatial profile of the derivative response at four consecutive time points (10–40 ms), for the example session in Fig. 5a. Because the evoked VSD response appeared close to the border of the imaging chamber, the spatial profile is not plotted symmetrically on both sides of peak response (location of peak response is denoted as 0 mm; the plotted distance is smaller for the closer border). Early times of the derivative response (10–20 ms) show a wide positive change of activation centered on the peak response, and lower values farther away from the peak. Later times (30–40 ms) showed negative values around the electrode site (gray arrow in Fig. 6a, LFS condition) while more remote sites showed values closer to zero (see 1p condition, t = 30 ms) or even positive values (black arrows in Fig. 6a, LFS condition, t = 30 ms). The negative derivative near the site of the microelectrode corresponds to the fast decline in the VSD signal, whereas the positive values at remote sites suggest the existence of an activation wave that is propagating laterally, starting near the electrode and spreading horizontally over the cortex.

To investigate the VSD signal propagation wave we performed a grand analysis of the spatial profile for HFS, LFS and HFS short conditions (Fig. 6b). The spatial profile of each session was aligned to peak response in space (at t = 10 ms) and normalized to the peak value at t = 10 ms of each session. At 30 ms, the grand analysis shows negative values near the electrode tip (gray arrow; p < 0.05, sign-ranked test for a significant difference from zero) while more remote sites showed positive values (black arrows; p < 0.05, sign-ranked test for a significant difference from zero).

**Figure 7:**
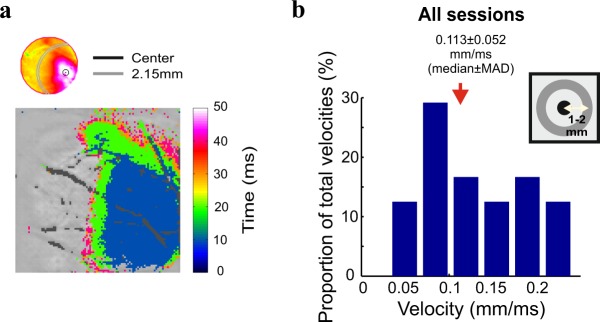
Propagation velocity. (**a**) Top: Illustration of two circular ROIs: black contour ROI positioned at the center of peak response, gray annulus ROI positioned at 2.15 mm from center. Bottom: Latency map denoting time to cross threshold of 30% from peak response for an example session. The latency map appears over the blood vessels image. Large blood vessels and electrode are colored in gray. (**b**) Distribution of propagation velocities over all sessions. The propagation velocity for each session was averaged over all rings. The red arrow denotes the median velocity across sessions. Inset: Schematic illustration of center and peripheral ROIs. The rings are centered on the peak activation in space. The peripheral ROI is located between 1.25 to 2.15 mm from the center.

To determine the propagation velocity of the VSD response, we selected a central ROI (circle, 5 pixels radius) located at the point of maximal activation in space and 7 non-overlapping peripheral rings extending 1.25–2.15 mm from the center (3 pixels width each, see Methods). We defined a threshold of 30% of peak response in each session and calculated for each ROI the time that the threshold was crossed (see Methods). Figure 7a shows a latency map i.e. the time at each pixel crossed the threshold for an example session. Pixels located closer to the peak response, passed that threshold earlier in time then remote pixels. Then, for each ring we calculated the velocity of propagation from the center (see Methods). Figure 7b shows the distribution of velocity calculated for all the rings from all sessions (0.113 ± 0.056 mm/ms, median ± MAD). The propagation velocity was similar for each individual condition (0.125 ± 0.07 mm/ms, 0.12 ± 0.03, 0.1 ± 0.02 and 0.15 ± 0.07 for 1p (n = 6), HFS (n = 6), LFS (n = 6) and HFS short (n = 6) respectively, median ± MAD; p > 0.05, Wilcoxon rank sum test). Similar velocity was obtained for a different threshold (0.134 ± 0.162 mm/ms median ± MAD; see Supplementary Fig. S4). The calculated velocity is within the reported range of horizontal connections propagation velocities which then suggests the involvement of horizontal connections it the response propagation.

### Comparison of the VSD response evoked by whisker deflection and ICMS

**Figure 8:**
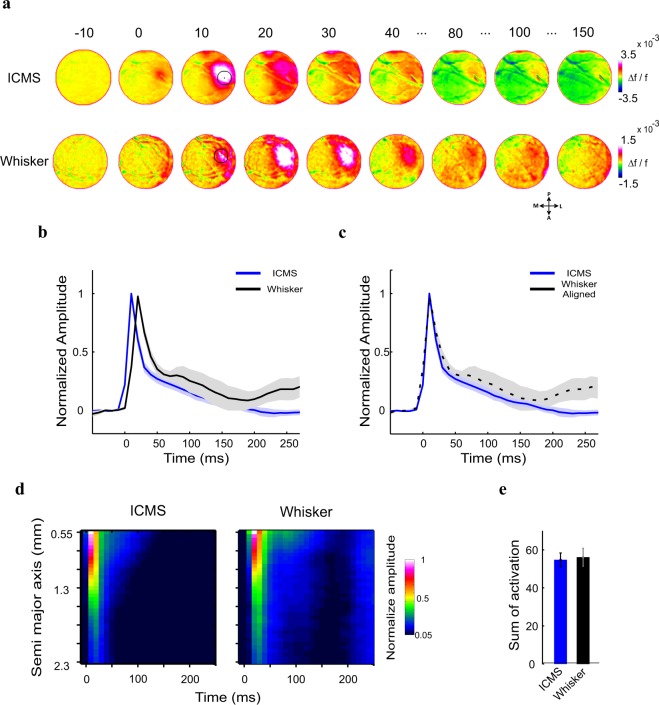
Population responses to one pulse ICMS and 1p whisker deflection are similar. (**a**) Example session: population response maps in the barrel cortex, evoked by 1p ICMS and 1p C2 whisker deflection (50 ms ramp-and-hold). The numbers above the maps show the time in ms after stimulus onset. The circle ROI (radius = 0.55 mm, black contour) centered on peak response is depicted on the map at t = 10. Maps are color coded. (**b**) Normalized time course of the VSD signal, averaged across sessions (on each condition the VSD response was normalized to peak response and then averaged across sessions). Shaded area represents ± 1 SEM over sessions (ICMS, n = 6; whisker, n = 9 sessions). (**c**) Same time course of the VSD responses as in b, after peak responses alignment. (**d**) Space vs. time plots: the VSD response at increasing distances from the response center as function of time. Response in each ellipse was normalized to the peak response (see Methods). (**e**) Sum of response activation over space and time (from t = 0 to t = 50 ms), for the maps depicted in d. Error bars represents ± 1 SEM over sessions. Wilcoxon rank-sum test: no significant difference (N.S).

Next we were interested to investigate the relation between the population response evoked physiologically by sensory stimulation or artificially by ICMS. Thus, we compared the VSD response evoked by a brief whisker deflection (see Methods) with 1p of ICMS (see Methods). Figure 8a shows data from an example session: a sequence of VSD maps evoked by 1p of ICMS (top row; same as in Fig. 2) and 1p of whisker deflection (bottom row). As expected, the evoked population response (at an ROI centered on the peak neural activation) following whisker deflection arrived to peak response later then the VSD signal evoked by ICMS (time to peak 20 ± 0 ms and 10 ± 0 ms for whisker deflection (n = 9) and ICMS 1p (n = 6) respectively; Fig. 8b). Interestingly, when aligning both responses on time of peak response (Fig. 8c), the VSD response dynamics of whisker deflection and ICMS showed high similarity. To compare the spread of cortical activity following whisker deflection or ICMS we applied an elliptical ring ROI analysis (see Fig. 4a and Methods). Figure 8d shows the space-time maps for the grand analysis where the VSD signal was normalized to peak response of each session. The maps show the neuronal population response as function of distance over cortical space (y-axis) along the semi major axis of the fitted ellipses for each time point (x-axis). The semi major axis of the central ellipse (located around peak response; see Methods) was: 0.55 ± 0.07 mm (mean across sessions ± sem), the semi major axis of the largest ellipse was: 2.3 ± 0.07 mm (mean across session). The spatio-temporal profile of the VSD response evoked by 1p ICMS showed similar activation over time and space compared with 1p whisker deflection (50 ms ramp-and-hold). To quantify this, we computed the sum of overall activation during whisker deflection (i.e. 0–50 ms from stimulation onset; see Methods) and over cortical space for each session. Figure 8e depicts the summed normalized activation, which is similar for the 1p ICMS and the whisker deflection (54.78 ± 3.6 and 56.22 ± 5.4 for 1p ICMS (n = 6) and whisker stimulation (n = 9) respectively; N.S: not significant, Wilcoxon rank sum test). These results are in accordance with previous studies that reported on similarities between the neural activation evoked by ICMS or whisker deflection^47,48^.

## Discussion

While ICMS is in use at research for many years, the effects of low and high frequency ICMS on the evoked spatio-temporal patterns of neural activity are not well understood. In this study we measured using VSDI the neural population response evoked by high (500 Hz) and a lower frequency (100 Hz) ICMS, in the barrel cortex of anesthetized rats. We found that ICMS at HFS was less effective in cortical activation on both the time and space domain, when compared to ICMS at LFS (although HFS has 5 fold more energy than the LFS). Furthermore, we showed evidence for a lateral propagation of the signal starting near the electrode site and spreading horizontally over the cortex. The calculated propagation velocity of the evoked pattern suggests the involvement of cortical horizontal connections.

The effects of ICMS on the evoked spatio-temporal pattern of neural activity or behavior depend on the electrical stimulation parameters. Previous electrophysiological, VSDI and optical imaging of intrinsic signal studies reported that increasing current amplitude lead to higher neuronal activity, extending over a larger brain area around the microelectrode site, including activity propagation to neighboring, interconnected cortical regions. Fehérvári *et al*. performed VSDI in mouse’s V1 while applying ICMS. They used variable current amplitudes (10–50 µA) and found that the VSD signal increased with larger current amplitude. In addition, the spatial spread of activation was limited for weaker stimulation, while at stronger stimulation, the evoked response appeared over a larger cortical area^33^. Similar results were observed when using optical imaging of intrinsic signal in anesthetized monkeys, in which higher currents (ranging from 10 to 200 µA) evoked larger cortical responses and recruited more cortical areas than lower currents^51^. In the latter study, the authors also manipulated the stimulation duration and found that the magnitude of the hemodynamic response increased with stimulation duration. The effects of current amplitude were tested at the behavioral level as well. For example, rats were trained to detect ICMS occurrence delivered to the barrel cortex and their detection performance increased with larger stimulation amplitude^29^. Frequency is another important parameter of ICMS, however its effects were mainly studied at the behavioral or motor level^28–30^. For example, Semprini *et al*. varied the frequency of the ICMS in rats trained to detect ICMS delivery to the barrel cortex and found that high detection rates were achieved with the range of 25–200 Hz. Thus little is known on how ICMS frequency shapes the evoked spatio-temporal pattern of neuronal activity.

Here we measured and quantified the effects of HFS and LFS ICMS on the evoked spatio-temporal patterns of neuronal responses and showed that LFS is superior over HFS in neural activation. LFS showed higher VSD signal that spread over a larger area during stimulation compared with HFS. The definition of LFS and HFS is quite variable among studies and depends on the stimulated tissue and the electrical stimulation technique. For example, studies of peripheral nerves stimulation defined HFS to be within the range of 1–40 kHz^38^ while studies using deep brain stimulation (DBS) defined HFS as 50 Hz^36^. In this work we used 500 Hz as HFS, that is well within the range of previous ICMS HFS studies^28,52,53^. As the frequency range for LFS is not well defined for ICMS, we chose 100 Hz as the lower frequency. The frequency range of 100–200 Hz was widely used in many previous studies and was reported to be highly effective, in evoking neural activity and affecting behavioral performance^28,52,54^.

Our results showed that the peak population response evoked by HFS or LFS appeared fast, within 10 ms post stimulation onset. This peak was followed by a fast descending phase and a sustained activity (Figs 2, 3 and 4) that persisted throughout the stimulation. This activity was lower for HFS than LFS. In addition LFS spread over larger cortical area than HFS. These results may suggest the involvement of an inhibitory effect in the HFS condition while another possibility is that HFS was less effective in directly activating the cortical tissue. Previous studies reported on evidences for involvement of GABAergic inhibitory neurons in sensory cortical processing, including in the barrel cortex, which share a proportional relation with the excitatory network. It was previously shown that frequency modulation of sensory input may lead to changes in the excitatory/inhibitory balance^55–58^ (for review see^56^). Short latency inhibition (fast time to peak, <10 ms)^56^ was suggested to be mediated via GABA_A_ inhibitory neurons which were shown to exist in layer 2/3^59^ as well as in layer 4^58^, the main layers for from which the VSD signal is collected. Therefore, it is possible that the observed early narrow peak response in LFS and HFS, followed by a fast decline and then a lower sustained activity in the HFS during train duration (100 ms; Fig. 3), arise from activation of this type of inhibitory neurons. The time scale of this modulation may be too early to be affected by GABA_B_ inhibitory neurons which are considered to have slower dynamics (recruited around 100–500 ms) mediated via G-protein^60^.

In addition to the above, it is also possible that HFS was less effective in recruiting excitatory responses within the cortical network. Few mechanisms were suggested in this relation. HFS can generate a conduction block which can lead to a lower neuronal activation at the site of stimulation. A conduction block may arise from an increased extracellular potassium concentration which can then lead to changes in neural excitability^36^ or reduction in the open probability of voltage-gated sodium channels^37,61^. In addition, Burrier *et al*. (2001) investigated HFS in the subthalamic nucleus (STN) in rats slices, *in-vitro*. They proposed that HFS-induced silence of neural activity was mediated by a reduction of Na^+^ and Ca^+2^ voltage-gated currents, which interrupted the ongoing activity of STN neurons^62^. In order to investigate the involvement of inhibitory processing that may underlie the response difference between LFS and HFS ICMS, additional studies are required.

An interesting aspect of ICMS technique is the ability to affect sensory perception and behavior in a specific manner. In our study and others^7,25^, there is evidence that even short trains of electrical stimulation are activating a large region of the cortex, larger than expected by passive spread of current and direct excitation of cortical elements^25^. These results raise the question: how can such a wide activation of cortical tissue lead to specific and precise behavioral effects? In a previous series of experiments performed in area MT of behaving monkeys Salzman *et al*. (1990, 1992) used low amplitude ICMS pulses (10 µA) at 200 Hz^27,63^ and reported on a specific behavioral effect. ICMS biased the monkey’s choices on a direction discrimination task towards the preferred direction of neurons at the stimulation site. Interestingly, a continues study of the same group^28^ showed that increasing the stimulation frequency to 500 Hz (at 10 µA) preserved the directional specificity of the microstimulation effect and increased the intensity of the directional signal, whereas using a higher current amplitude at lower frequencies (80 µA, 200 Hz) reduced or eliminated the behavioral effect. The interpretation of these results can be explained with larger spread of neural activation around the electrode at lower frequencies and high stimulation currents, whereas for higher frequencies, the evoked activity was spatially restricted to neurons having similar preferred direction. Indeed increasing the current amplitude alone, induced a larger spread of the neuronal activity within and across cortical areas^32,33^. The above behavioral and neuronal results and the interpretation are in accordance with our observation, that HFS induces cortical activation that spread to a smaller cortical region, even at high current amplitudes (i.e. 80 µA), and thus may cause a more spatially restricted activation, within the columnar range.

The high spatio-temporal resolution of the VSDI technique offers the opportunity to image activity dynamics over milliseconds at the mesoscale resolution, following stimulation and to investigate activity propagation and cortical connectivity. We used a ring analysis (Fig. 7) to determine the propagation velocity of the ICMS evoked response. Using this approach we computed the propagation velocity of the VSD response and the median value was 0.113 ± 0.052 mm/ms. This velocity is in accordance with previous VSD reports of lateral propagation velocities, from upper cortical layers of rodent *in-vitro*^64–66^ and *in-vivo*^50^ although the calculation method and the stimulation parameters were different. The similarity of horizontal spread velocity among several studies suggests that ICMS can be used to reveal characteristics of the underling cortical connectivity network.

ICMS provides a way for artificially activating the cortical network by directly activation neurons and axons passing near the stimulating electrode. Neural activation can then spread and propagate through the activated network connectivity. Previous studies reported that ICMS can mimic the neural response evoked by sensory stimulation^50,67,68^ and can also evoke natural behavior^69,70^. These reported observations are consistent with our results, showing that the evoked population response of 1p ICMS resembles the evoked response following brief whisker deflection. The similarity is evident for both the spatial spread and response dynamics (Fig. 8). Partial explanation for the high similarity might be the fast dynamics of both whisker deflection (by the piazo actuator) and 1p ICMS (current spread of the short single pulse).

In the absence of external stimulation, primary sensory cortices, show spontaneous activity patterns and propagation that resembles cortical activation evoked by sensory stimulation^71–74^. Using photostimulation Mohajerani *et al*. (2013), showed that they can induce activation patterns similar to those observed in spontaneous activity or sensory stimulation^73^. Moreover, a recent study by Carrillo-Reid *et al*. (2016) showed that repetitive two-photon photostimulation in sensory cortex, can build and imprint cortical ensembles that recur spontaneously. Interestingly, the spontaneous activity mimics the repetitive photostimulated response. Future studies are needed to investigate whether ICMS can also imprint new cortical ensembles.

In conclusion, we found that HFS of ICMS was less effective in cortical activation compared to the LFS condition which may result from a suppression of axonal conduction during HFS, the involvement of inhibitory network or both. However, we note that our observations are limited to stimulation in the upper layers (250–400 µm) of the barrel cortex and additional studies are required to uncover whether there are layer specific stimulation effects.

## Materials and Methods

### Animals and surgical procedure

Six male albino rats (Sprague Dawley (SD), 200–350 gr) were used for the experiments and data analysis. All experimental and surgical procedures were carried out according to the NIH guidelines, approved by the Animal Care and Use Guidelines Committee of Bar-Ilan University and supervised by the Israeli authorities for animal experiments. Rats were deeply anesthetized with Urethane (1.5 gr/kg), which provides a long lasting stable anesthesia. A craniotomy was performed above the barrel cortex area of the rat (stereotactic coordinates: 2 mm posterior and 6 mm lateral to the bregma) and the dura mater was carefully removed in order to expose a ~5 mm × 10 mm window over the barrel cortex.

### Voltage-sensitive dye staining and imaging

A staining chamber was used in order to stain the exposed cortex with voltage sensitive dye (RH-1838; 0.5 mg/ml of artificial cerebrospinal fluid (ACSF) for ~2 hours. Following staining, the brain was washed with ACSF solution, covered with agarose and sealed with a custom cut coverslip. For voltage-sensitive dye imaging (VSDI) we used the MicamUltima system and images of 100 × 100 pixels (the whole image covers an area of 5^2^ mm^2^; each pixel cover cortical area of 50^2^ μm^2^) were acquired at 100 Hz. During imaging, the exposed cortex was illuminated using an epi-illumination stage with an appropriate excitation filter (peak transmission 630 nm, width at half height 10 nm) and a dichroic mirror (DRLP 650 nm), both from Omega Optical, Brattleboro, VT, USA. In order to collect the fluorescence and reject stray excitation light, barrier post-filter was placed above the diachronic mirror (RG 665 nm, Schott, Mainz, Germany; Fig. 1a). To obtain the vascular pattern of the imaged area we used a green light (540 nm bp10). Next, VSDI was performed for the next 2–3 hours.

### Whisker stimulation and mapping the barrel fields

To verify that we are measuring neuronal population responses from the barrel cortex (in addition to cortical exposure at the adequate anatomical coordinates, see above), different individual whiskers (e.g. B2 or C2) were deflected separately by a piezoelectric stimulator. Each whisker was glued to a thin glass pipette attached to the piezoelectric device ~5 mm from the whisker’s base and was deflected along the anterior-posterior axis of the head (1–5 pulses; pulse duration: 50 ms; frequency: 10 Hz). The piezo bending actuator reaches its nominal displacement within ~0.5 µs. Then the cortical maps were obtained using VSDI (Fig. 1a). To evaluate the barrel field size and define the different barrels, we used the activated area at early times (10 ms after stimulus onset). Pixels exceeding a high response threshold (75% of peak activity within the barrel cortex) were included in the region of interest (ROI). This threshold reconciled well with the expected size of a single barrel field as shown in previous studies using a similar approach^47^. Figure 1b left shows an example of the VSD response pattern that is corresponding to C2 barrel field following C2 whisker deflection (1p, 50 ms duration). The map in Fig. 1b top represents the pattern of activation 10 ms after stimulation onset while C2 barrel field is depicted by a black contour (75% of maximal response amplitude). Figure 1b bottom shows the outline of different barrel fields over the image of the blood vessels pattern. This map enables to obtain the rows and columns location in the barrel field. Based this stage we could target the microstimulation electrode to the barrel cortex.

### Intra-cortical microstimulation parameters

A microelectrode was targeted to the barrel cortex (identified by the evoked responses to whisker stimulation; Fig. 2a) and inserted in the upper layers (250–400 µm; L2/3). Biphasic square pulses were delivered through a standard tungsten microelectrode (FHC, Bowdoin, ME, USA) using a microstimulation box (linear biphasic stimulus isolator, BAK electronics, BSI-1A). Each biphasic pulse is composed from a cathodal (0.2 ms) pulse followed by an anodal (0.2 ms) pulse (Fig. 1c). We stimulated the brain with current amplitude of 80 µA in 4 different stimulation conditions: single-pulse stimulation (1p; Fig. 1c top), low-frequency stimulation (LFS; 100 Hz; 10 pulses, 100 ms duration; Fig. 1c middle) and two high frequency conditions of different duration lengths: (i) 100 ms of high-frequency stimulation (HFS; 500 Hz; 50 pulses; Fig. 1c bottom), in this condition stimulation length equals the LSF, but it has X5 energy. (ii) 20 ms of high-frequency stimulation (HFS short; 500 Hz; 10 pulses), in this condition, the amount of energy equals to that of LFS, but stimulation length is much shorter. ICMS frequency definition for HFS and LFS has a wide range and depends on stimulated tissue and the electrical stimulation technique; In this work we used 500 Hz as HFS, that is well within the range of previous ICMS HFS studies^28,52,53^. Because the frequency range for LFS is not well defined for ICMS, we chose 100 Hz as the lower frequency. The frequency range of 100–200 Hz was widely used in many previous studies and was reported to be highly effective, in evoking neural activity and affecting behavioral performance^28,52,54^. We used additional control stimulation condition: 5 pulses of lower frequency (50 Hz) that has half of the LFS energy but equal time duration (100 ms). The output current from the microstimulation box was verified as voltage measurement across a 100 KΩ resistor located between the animal and the microstimulation box.

### Data analysis

Data analysis was performed on 18 recording sessions of stimulation conditions and 13 control sessions from 6 rats.

### Basic VSDI analysis

All data analyses and calculations were done using MATLAB software. The basic analysis of the VSD signal is detailed elsewhere^75–77^. Briefly, to remove the background fluorescence levels, each pixel was normalized to its baseline fluorescence level (average over first few frames, before stimulation onset). The heart beat artifact and the photo bleaching effect were removed by subtraction of the average of blank signal (recorded in absence of stimulation) from stimulated trials. Thus, the imaged signal (Δf/f) reflects relative changes in fluorescence compared to the resting level observed at blank trials. For further analysis, VSD maps were computed by averaging over all trials in a single condition.

### Defining regions of interests (ROIs)

To study the spatial and temporal properties of the VSD signal in a given area, ROIs were defined. ICMS evoked neuronal population responses first near the electrode tip which then spread across the cortical surface. For each location of the microelectrode, a circle ROI, radius of 11 pixels (0.55 mm, 373 pixels total) was set at the peak spatial location of the VSD response. Thus, the same ROI was used for different stimulation conditions, but with the same microelectrode position. By averaging the VSD signal over pixels in the desired ROI we obtained the time course of response. In most experiments, the peak VSD response activation is slightly shifted from the microelectrode penetration site (Fig. 2a) due to the microelectrode sharp penetration angle (limited by the vicinity of large optical lenses).

### Time to half peak response

To study the temporal characteristics of HFS and LFS we calculated the time to half peak response at the rising phase and descending phase of the response. To compute accurate time-to-desired threshold, we applied a linear fit to the rising phase of the response, and calculated the point at which it crossed the absolute threshold. This enabled us to overcome the temporal limitation imposed by our sampling rate (100 Hz, 10 ms per frame). Since the descending phase showed nonlinear pattern, mainly for HFS and LFS condition, we applied this approach only at the rising phase.

### Space time analysis (elliptical ring ROI analysis)

Space time analysis was applied in order to quantify and compare the spatial profile spread of the VSD signal. We generated a continuous set of non-overlapping elliptical shape rings (see schematic illustration in Fig. 4a), centered over the spatial peak of the evoked response and fitted to the activation pattern at 10 ms post stimulation. The size of the major axis and minor axis of the ‘ellipses’ changed from the fitted ellipse at steps of 50 µm (one pixel) for each ring to create a set of 40 consequential elliptical rings. The central ellipse was defined at the 5^th^ elliptical ROI and the VSD response of the central ellipse was defined to be mean across ring no. 1–5, to pass a threshold of minimal number of pixels. The central ellipse was located around the activation peak response.

### Derivative maps

To study the propagation dynamics of the evoked VSD signal across cortical surface we computed derivative maps that were defined by Equation (1), where t is time in ms:

1 $$\mathrm{derivative}\,\,{\rm{map}}\,({\rm{time}}={\rm{t}})={\rm{map}}({\rm{t}})-{\rm{map}}({\rm{t}}-20)$$

### Propagation velocity calculation

To calculate the velocity of response propagation from ICMS site across cortical surface, we defined for each session the following ROIs: a center ROI located at the point of peak activation in space (circle, 5 pixels radius) and 7 peripheral ring (each of 3 pixels width) ROIs, co-centered with the center ROI, of increasing radius from 1.25 to 2.15 mm. We then defined a threshold as 30% of peak VSD response in each session, and calculated for each ROI the time in which the VSD signal passed the threshold. Finally, we calculated for each ring the velocity of propagation from the center as follows:

2 $${\rm{v}}=\frac{{\rm{\Delta }}x\,({\rm{mm}})}{{\rm{\Delta }}t\,({\rm{ms}})}$$

where Δx is the distance of the ring from the center and Δt is the difference in time-to-threshold between the ring and center ROIs. To compute accurate time-to-threshold, we applied a linear fit to the rising phase of the response, and calculated the point at which it crossed the absolute threshold. This enabled us to overcome the temporal limitation imposed by our sampling rate (100 Hz, 10 ms per frame).

### Statistical analysis

To compare the VSD response across stimulation conditions, we used nonparametric Wilcoxon Rank-Sum test and applied Bonferoni correction for multiple comparisons. Data are presented as mean ± SEM or median ± MAD.

## Electronic supplementary material

Supplementary Figures S1–4
