# Supplementary material for: Spatio-temporal characteristics of population responses evoked by microstimulation in the barrel cortex
Source: Sci Rep. 2018 Sep 17;8:13913. doi: 10.1038/s41598-018-32148-0 (PMC6141467; doi:10.1038/s41598-018-32148-0)
Supplement: Supplementary file 1 — Supplementary Figures S1–4 [file 41598_2018_32148_MOESM1_ESM.docx]

**Supplementary information**

**Title:**

Spatio-temporal characteristics of population responses evoked by microstimulation in the barrel cortex

**Authors:**

Shany Nivinsky Margalit *^1^, Hamutal Slovin^1^

**Affiliation:**

1. The Gonda Multidisciplinary Brain Research Center, Bar-Ilan University, Ramat Gan, 52900 Israel

**Supplementary Figures S1-4**

**Figure S1**


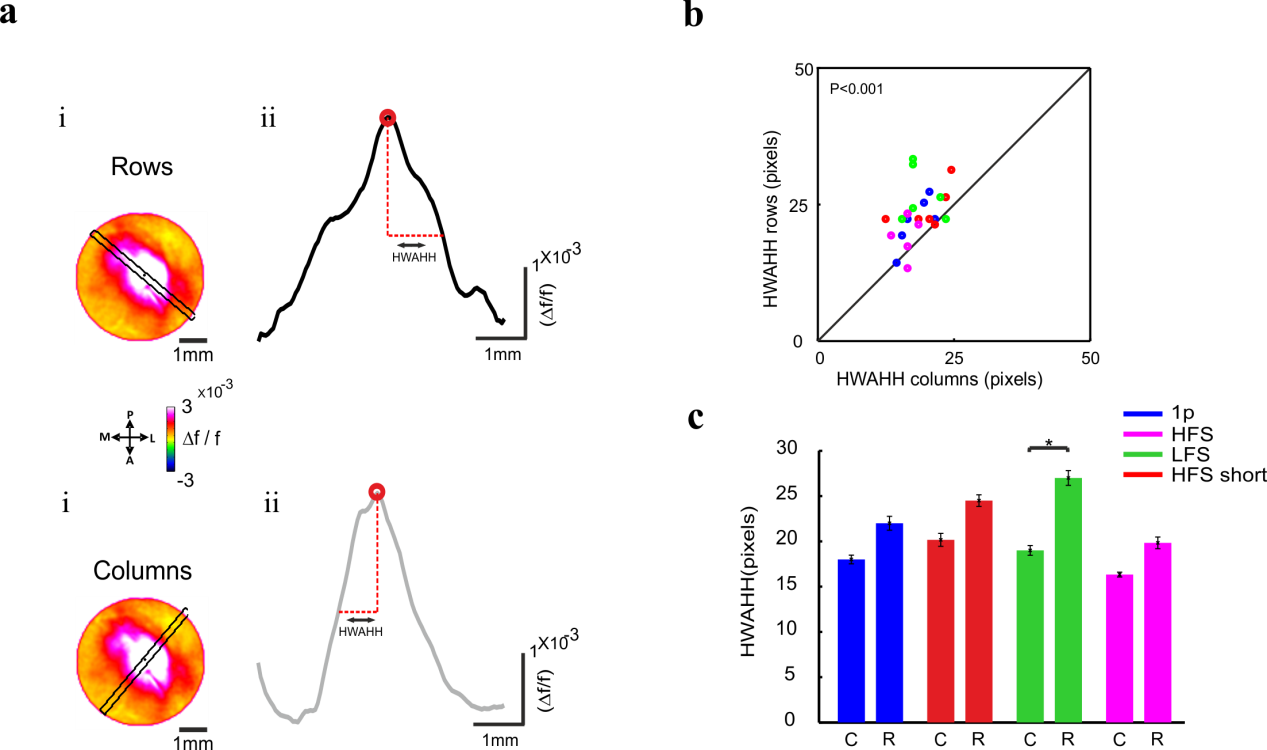


**Figure S1. Anisotropic spatial spread in the barrel field.** **ai**: Illustration of a spatial profile (250 µm width) along the rows in the barrel field (top) and the columns (bottom) for LFS example session. The profile is passing through peak response in space. **aii**: Spatial profile curves computed for peak response in time (averaged across ±10 ms). The red dot denotes the peak of the spatial profile, while the red dashed line is the half width at half height of the peak response (HWAHH). **b:** Grand analysis of HWAHH in rows vs. columns, over all recording sessions. Wilcoxon rank-sum test: p<0.001. Each pixel is 50^2^ µm. **c**: Mean HWAHH for the rows (’R’) and columns (’C’) profiles for 1p, HFS, LFS and HFS short. Error bars are one SEM over sessions (1p, n=6; HFS, n=6; LFS, n=6; HFS short, n=6)


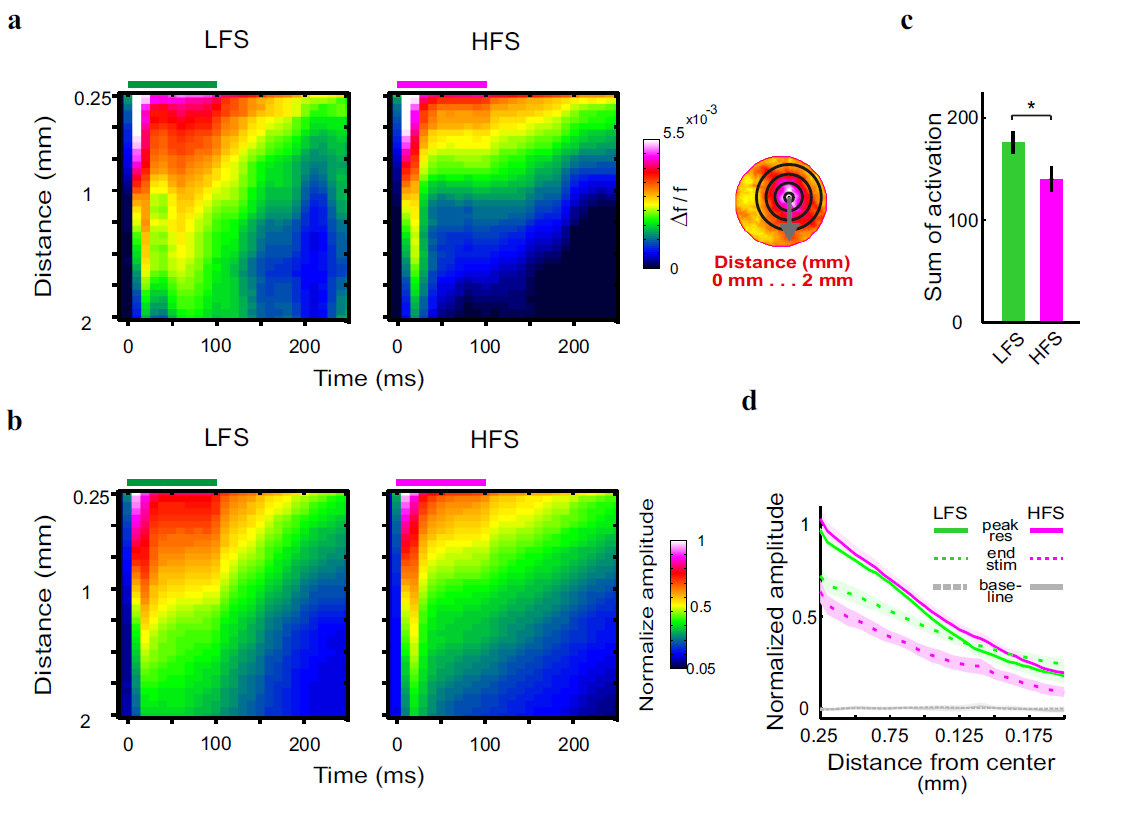
**Figure S2**

**Figure S2. Space time analysis using ring ROIs. a-b**. Space vs. time plots: the VSD response at increasing distances from the center as function of time. Response in each ring was normalized to the mean peak response of the five first rings in the LFS condition (see Methods). Horizontal bars at the top represent the stimulation duration for an example session (a left; same as at Fig. 2) and for grand average (b). a right: Schematic illustration of ring shape ROIs. The radius of the successive rings (that are co-centered on the peak activation in space), is increased by one pixel (50 μm) width. The largest ring has a radius of 2000 μm. Zero distance refers to the peak activation. **c**: Sum of response activation over space and time (from t=0 to t=100 ms), for the maps depicted in B. Error bars represents ±1 SEM over sessions. Wilcoxon rank-sum test: * p<0.05. **d**: Spatial profile of the responses, across all sessions, at time of peak response (peak res, solid line) and when stimulation was ended (end stim, dashed line). Red lines represent the baseline activity for HFS (solid line) and LFS (dashed line). The profile is normalized to the mean peak response across the five first rings of LFS. The shaded area is ±1 SEM over sessions (LFS, n=6; HFS, n=6 sessions).

**Figure S3**


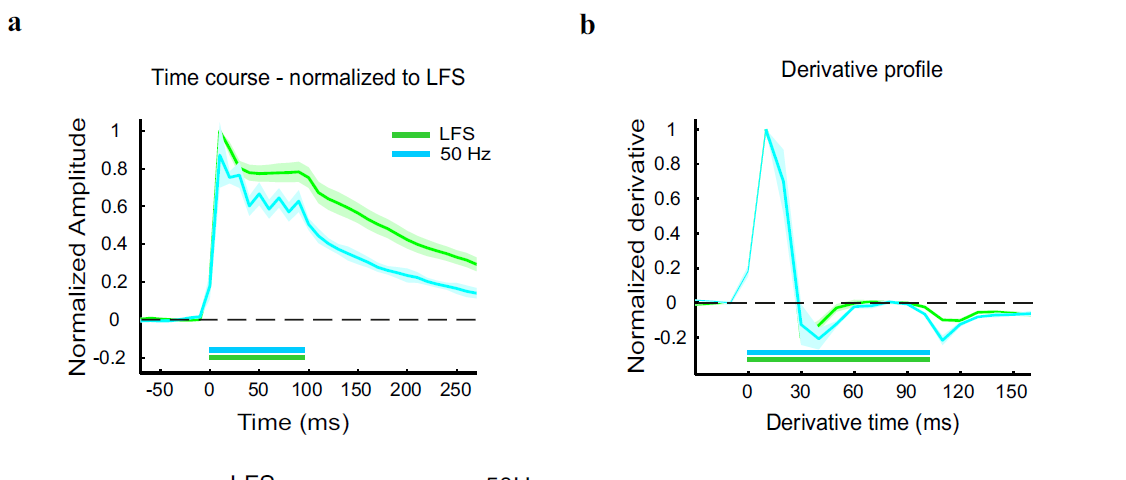

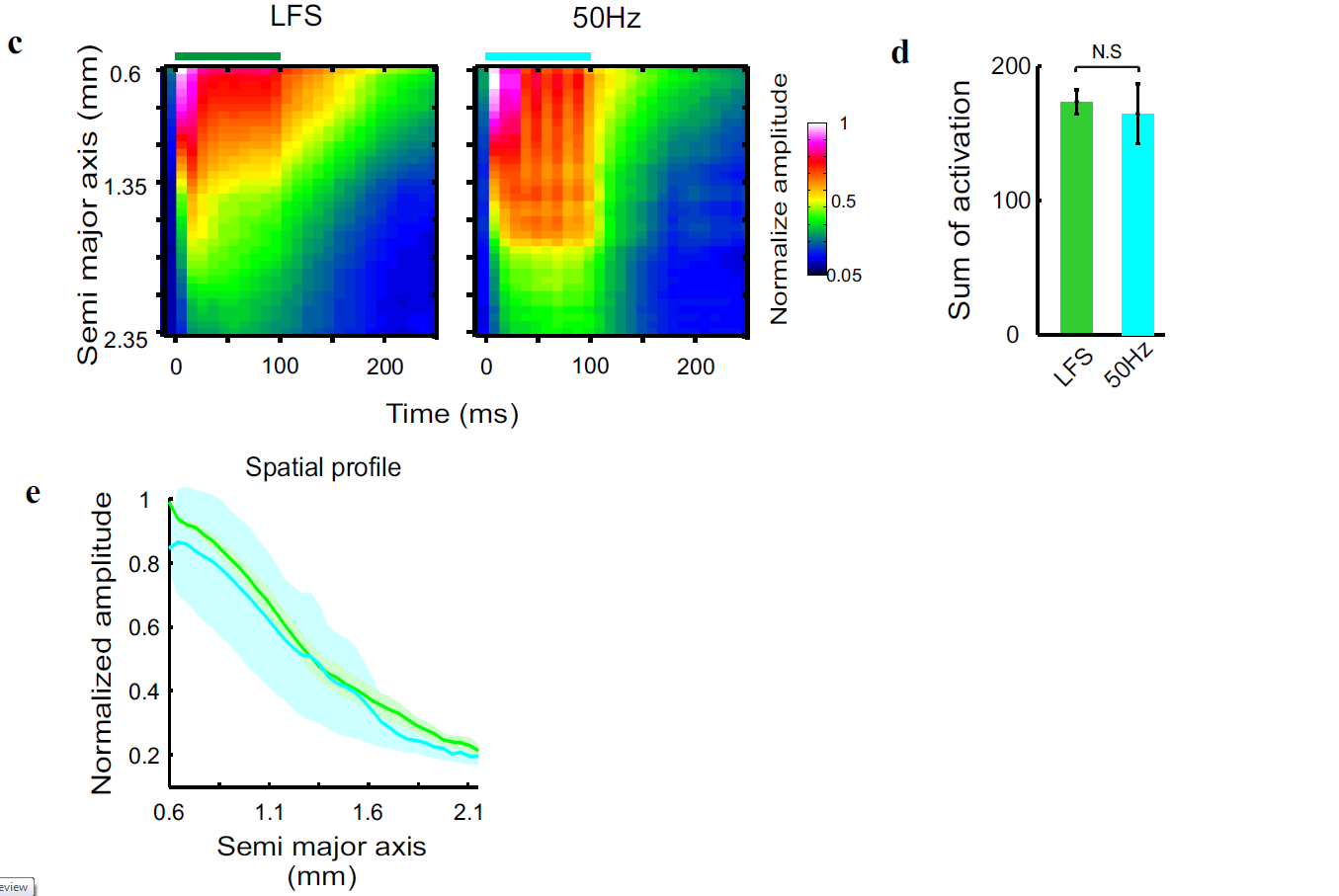


**Figure S3.** **Population response to 50Hz stimulation and comparison to LFS.** **a**: Grand average normalized time course of the VSD signal evoked by 50 Hz or 100 Hz (LFS). Time course in each session was normalized to peak response of the LFS condition and then averaged across all sessions. The shaded area is ±1 SEM over sessions. Horizontal color bars refer to ICMS duration. **b**: Normalized derivative response averaged across sessions (the VSD response was normalized to LFS peak response). **c**: Space vs. time plots: the response at increasing distances from the center as function of time. Same as in Figure 4 (see Methods). Horizontal bars at the top represent the stimulation duration. **d**: Sum of response activation over space and time (from t=0 to t=100 ms), for the maps depicted in c. Error bars represents ±1SEM over sessions. Wilcoxon rank-sum test: no significant difference (N.S). **e:** Spatial profile of the responses, across all sessions at time of peak response. The profile is normalized to the mean peak response across the four first rings of LFS. The shaded area is ±1 SEM over sessions (LFS, n=6; 50 Hz, n=2).

**Figure S**
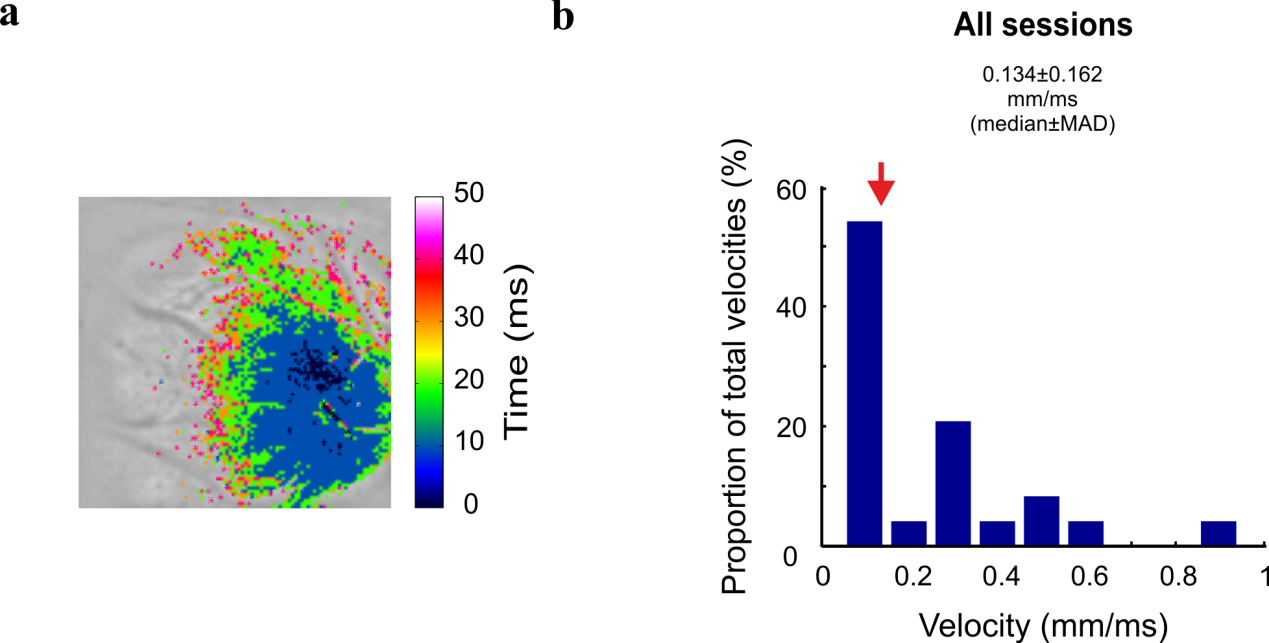
**4**

**Figure S4. Propagation velocity using a SNR approach**. **a**: Latency map denoting time to cross threshold of 5 times STD from mean activity in baseline. The latency map appears over the blood vessels image. **b**: Distribution of propagation velocities over all sessions. The propagation velocity for each session was averaged over all rings. The red arrow denotes the median velocity ± MAD across sessions. The results are similar to those shown in Fig. 7b.
